# Core-genome multilocus sequence typing and core-SNP analysis of *Clostridium neonatale* strains isolated in different spatio-temporal settings

**DOI:** 10.1128/spectrum.02766-23

**Published:** 2023-11-01

**Authors:** Victoria Mesa, Johanne Delannoy, Laurent Ferraris, Laure Diancourt, Christelle Mazuet, Frédéric Barbut, Julio Aires

**Affiliations:** 1 Université Paris Cité, INSERM, UMR-S 1139 (3PHM), Faculté de Pharmacie de Paris, Paris, France; 2 Institut Pasteur, Université de Paris Cité, Centre National de Référence des Bactéries anaérobies et Botulisme, Paris, France; University Paris-Saclay, AP-HP Hôpital Antoine Béclère, Clamart, France

**Keywords:** *Clostridium neonatale*, necrotizing enterocolitis, NEC, wgMLST, cgMLST, cgSNP

## Abstract

*Clostridium neonatale* was described as a new species within the *Clostridium* genus cluster I *sensu stricto*. It was recovered from the gut microbiota of infants and has been associated with necrotizing enterocolitis (NEC), a severe gastrointestinal disease affecting preterm neonates. In the absence of molecular typing methods, we developed a core (cg)-genome multilocus sequence typing (MLST) scheme based on 48 newly sequenced and 12 publicly available genomes of *C. neonatale*. Using the ChewBBACA algorithm, a stable MLST scheme consisting of 2,350 target genes (with genomes having ≥95% cgMLST targets) is proposed. The strains are distributed among five clades, and while some are clonality related within clades, the *C. neonatale* strain distribution is independent of geographic or temporal clustering. When considering strains isolated from patients with and without NEC, there were no observable differences in clustering. When compared to a core-single nucleotide polymorphism (SNP) analysis approach (31,248 positions), we showed that both cgMLST and cgSNP have comparable genetic discrimination of strains. In this study, we validated the use of cgMLST and cgSNP-based typing methods for the genetic comparison of clinical isolates of *C. neonatale*, which will allow for future surveillance and epidemiological clinical investigations of this potential opportunistic pathogen.

**IMPORTANCE:**

*Clostridium neonatale* has been isolated from the fecal samples of asymptomatic neonates and cases of necrotizing enterocolitis (NEC). Taking advantage of a large collection of independent strains isolated from different spatio-temporal settings, we developed and established a cgMLST scheme for the molecular typing of *C. neonatale*. Both the cgMLST and cgSNP methods demonstrate comparable discrimination power. Results indicate geographic- and temporal- independent clustering of *C. neonatale* NEC-associated strains. No specific cgMLST clade of *C. neonatale* was genetically associated with NEC.

## INTRODUCTION

Necrotizing enterocolitis (NEC) is one of the most frequent neonatal intestinal diseases occurring in extremely preterm neonates. The NEC incidence ranges from 3% to 15% in high-income countries and is associated with a high mortality rate and long-term complications (1). *Clostridium neonatale*, a Gram-positive rod with subterminal spores that is strictly anaerobic and mobile, was first isolated from the fecal samples of neonates during a 1999–2000 outbreak of NEC in a Canadian neonatal intensive care unit (NICU) (2). Since 2002, *C. neonatale* has been associated with NEC in multicenter (3) and regional (4) studies as well as in a case report (5). In addition, a few studies have isolated *C. neonatale* in fecal samples of neonates without symptoms (6 – 10), suggesting an asymptomatic carriage.

Although briefly described in 2002, the classification of *C. neonatale* as a new species was not formally published. In 2014, based on a polyphasic approach, *C. neonatale* was proposed to represent a new species within the *Clostridium* genus (7). The name and species of *C. neonatale* were validated within the *Clostridium* genus cluster I *sensu stricto* in 2018 (11). The ambiguous status of *C. neonatale* between 2002 and 2018 could have led to misidentification and/or inadequate representation of *C. neonatale* populations in prior studies. Consequently, very little is known about *C. neonatale*’s genetics, population structure, or evolution. Recently, we published the complete genome of the *C. neonatale* reference strain 250.09 (= ATCC BAA-265^T^) and conducted a comparative genome analysis with eight available draft genomes that were accessible. We found that *C. neonatale* possesses an open pan-genome with genetic diversity and a flexible gene repertoire (12). In terms of molecular typing tools, a multilocus sequence analysis (MLSA) approach was used to show that *C. neonatale* belonged to the *Clostridium* genus *sensu stricto* (7). Furthermore, a quantitative real-time PCR targeting the *rpoB* gene was developed to detect *C. neonatale* from the fecal samples of patient (4).

The development of next-generation sequencing as a cost-effective technology has enabled genomic epidemiology monitoring and source tracking (13). This noticeably increases the amount of information available to compare bacterial strains by improving the discriminatory power of bacterial typing. In particular, the core-genome MLST (cgMLST) and core-single nucleotide polymorphisms (cgSNP) methods are largely used for bacterial typing and epidemiological analysis purposes (14 – 17). Relying upon whole genome sequencing, cgMLST extends the classical MLST concept to include the genes that make up the bacterial core genome, resulting in a systematic allele numbering system (14). cgMLST is considered to be highly discriminatory and less susceptible to deletions and other mutations in the genome (13). The cgSNP variant calling approach is another suitable typing method which can provide greater discrimination than cgMLST. It uses a representative reference genome and allows for the filtering of recombinant regions (18).

To the best of our knowledge, neither the cgMLST scheme nor the cgSNP approach has been developed or applied to *C. neonatale*. In this study, taking advantage of a unique well-characterized strain collection, we first created an *ad hoc* cgMLST scheme using the open source ChewBBACA algorithm (19). The scheme is developed using 48 newly sequenced *C. neonatale* and 12 publicly available genomes at the time of this publication. Second, both cgMLST and cgSNP methods were used to investigate the epidemiological phylogeny and genetic relationships of *C. neonatale* at the strain level. Additionally, the cgMLST and cgSNP methods were employed to distinguish strains obtained from NEC cases and controls in different spatio-temporal settings.

## MATERIALS AND METHODS

### Strain origin

**Fig 1:**
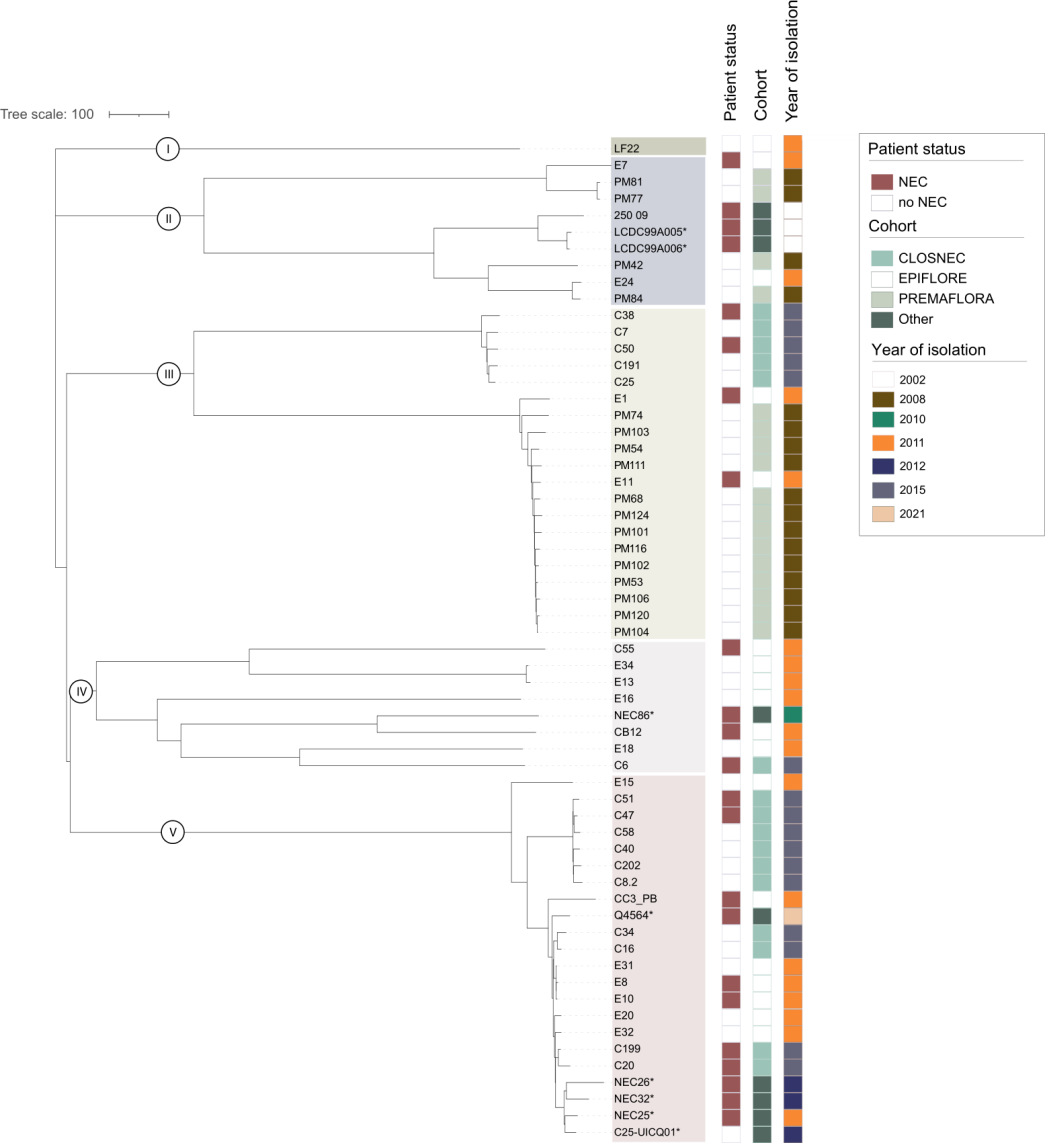
Neighbor-joining cgMLST phylogenetic tree of the 60 *C. neonatale* strains. Strain ID, patient status (NEC case or control), cohort, and year of isolation are given for each strain. The *C. neonatale* type strain 250.09 is the reference strain. (*) Strains from other studies (4, 5).

All strains were previously isolated from the fecal samples of neonates enrolled in three clinical studies (Fig. 1). The PREMAFLORA cohort (ANR-07-PNRA-007) included infants less than 37 weeks of gestational age who were hospitalized at a French NICU between 2008 and 2009 (20). The EPIFLORE study took place in 2011, and the ClosNEC study was conducted between 2015 and 2016. These were NEC case-control multicenter studies that involved preterm neonates less than 32 weeks of gestational age in 12 French NICUs (3, 21). NEC cases and control PN were obtained from the same NICU and were matched for gestational age, birth weight, type of feeding, and mode of delivery. Each NEC case was matched with two controls. The presence of clinical evidence fulfilling the modified criteria for NEC Bell’s stage II (associated with radiologic pneumatosis intestinalis) or III (definitive intestinal necrosis seen at surgery or autopsy) confirmed the diagnosis of NEC as defined by the neonatal clinical team (1). No outbreak was declared during the inclusion periods. All studies were conducted in accordance with the relevant French guidelines and regulations, and informed consent was obtained from the parents of all enrolled children. Of note, in 2008–2009, parental consent was sufficient to ensure that fecal samples were collected under ethical conditions. The EPIFLORE and ClosNEC cohorts (clinical trial nos. NCT01127698 and NCT02444624, respectively) were approved (nos. 911009 and 915094, respectively) by Commission Nationale de l’Informatique et des Libertés and the Consultative Committee on the Treatment of Information on Personal Health Data for Research Purposes (approval nos. 10.626 and 15.055, respectively).

### Strain isolation, media, and growth conditions

For the EPIFLORE and ClosNEC cohorts, stool samples were collected at the time of NEC diagnosis (the first stool was collected after the diagnosis). For neonates in the control group, fecal samples were collected within 1 week after their NEC cases. Strains were previously isolated from fresh stool samples collected from diapers, placed into sterile tubes containing 0.5 mL of brain-heart infusion broth supplemented with 20% glycerol (a cryoprotective agent), and immediately frozen at −80°C. For isolation of *C. neonatale*, the fecal samples were crushed in the brain-heart infusion broth with an Ultra-Turrax T25 (Fisher Bioblock, Illkirch, France). They were then diluted in peptone water, and 10^–2^, 10^–4^, and 10^–6^ dilutions were spread on a sulfite-polymyxin-milk selective agar medium (22) with the use of a WASP apparatus (Don Whitley Scientific, UK). After incubation under anaerobic conditions (CO_2_:H_2_:N_2_, 10:10:80) in an anaerobic chamber (Don Whitley Scientific), colonies were identified using matrix-assisted laser desorption ionization-time of flight mass spectrometry (MALDI-TOF MS, Bruker Daltonics S. A.). When MALDI-TOF MS identification was inconclusive, amplification and sequencing of the 16S rDNA gene were performed as described previously (7). Bacterial counts were reported as log_10_ colony forming units (CFU)/g of feces, with a count threshold of log_10_ CFU/g of feces. The reference strain 250.09 (= ATCC BAA-264^T^ = CCUG 46077^T^) was also included in the study (12). All *C. neonatale* strains were subcultured in a liquid TGYH broth (tryptone 30 g/L, glucose 5 g/L, yeast extract 20 g/L, and hemin 5 mg/L) or TGYH agar media and incubated under anaerobic conditions for 48 h at 37°C.

### Whole genome sequencing

Genomic DNA was extracted from 24 h bacterial liquid cultures using the DNeasy UltraClean microbial kit (Qiagen, Courtaboeuf, France). Sequencing of the 48 new *C. neonatale* genomes included in the present study was carried out by the Biomics sequencing platform (Institute Pasteur, Paris, France) using the Illumina Nextera XT DNA Library Prep kit and HiSeq or NextSeq 500 system sequencing devices. Paired-end reads were preprocessed using fqCleanER v.3.0 (https://gitlab.pasteur.fr/GIPhy/fqCleanER) with default parameters. The *de novo* assembly of the 48 genomes was performed using SPAdes v3.15.4 (23) with *k-mer* lengths of 21, 33, 55, and 77. The assembled contigs were evaluated for quality using QUAST. The genome annotation for the reference strain *C. neonatale* 250.09 (GenBank accession no. SAMEA9534266) was transferred to 48 genomes with 80% of similarity and identity values using the MicroScope pipeline platform v3.16.0, as previously reported (12). The CheckM (v1.2.2) marker gene set was used to evaluate completeness and contamination in each genome (24).

### Genome similarities

Genome similarity was measured using the FastANI tool (25) (last accessed 3 March 2022), which computes a pair-wise average nucleotide identity (ANI) value for the sample genome based on BLAST (ANIb). The resulting matrix was visualized using R package heatmap software from R package (26).

### Development and creation of the *C. neonatale* cgMLST scheme

The *C. neonatale* cgMLST scheme was established using the Blast Score Ratio-Based Allele Calling Algorithm workflow v.2.7.0 (chewBBACA) (19). First, a wgMLST scheme was constructed containing each coding DNA sequence (CDS) of the 60 strain genomes included in the present study. This step was performed by the *CreateSchema* operation, during which Prodigal v2.6.3 (27) identified each CDS. The CDS comparison (pairwise) and an all-against-all BLASTP search enable the grouping of genes that code for equivalent or very similar proteins (default blast score ratio above 0.6) as alleles for the same locus and catalog them in a unique file. This procedure defines the scheme as a set of CDS, each representing a single allele at distinct loci. Second, based on the obtained CDS file, the *AlleleCall* operation identifies paralogous loci from the obtained CDS file, which are then excluded using the *RemoveGenes* module. The resulting list of loci corresponds to the wgMLST scheme from which the cgMLST scheme is extracted. Third, the quality of the wgMLST scheme was evaluated using the *TestGenomeQuality* algorithm with 100 and 200 thresholds prior to extracting the cgMLST scheme (Fig. S1). This step investigates how threshold values affect the number of loci in the cgMLST scheme in relation to the number of genomes included. To extract the cgMLST, we utilized the *ExtractCgMLST* module and conducted a core loci polymorphism analysis with the *SchemaEvaluator* module using default parameters. Allelic profiles of the core locus, shared by 95% of the analyzed isolates, were utilized to generate a cgMLST similarity tree. The neighbor-joining algorithm (StandardNJ) was employed in the GrapeTree software v.1.5.0 (28) using the FastME implementation. Trees were edited using the iTOL v.6.5.7 tool (29). Furthermore, a minimum spanning tree based on the cgMLST allelic profiles of the 60 *C. neonatale* strains was created using GrapeTree.

### Core-SNP-based phylogeny

The cgSNP analysis was conducted on the raw data of 52 genomes. Since we did not have access to the initial raw sequencing data before reassembly, we excluded C25-UICQ01, NEC25, NEC26, NEC32, NEC86, LCDC no.99-A-005, LCDC no.99-A-006, and Q4564 genomes from the cgSNP analysis. The complete genome of the reference strain *C. neonatale* 250.09 served as the reference for the read mapping and cgSNP variant calling. The cgSNP analyses were performed using Snippy v4.6 (30) with default settings. The resulting file underwent filtering of variants with high densities of base substitutions, which were identified as possible recombination events with default parameters set in Gubbins version 3.0.0 (30). This process generated a recombination-free core-genome alignment. RAxML-NG was employed to develop a maximum likelihood tree with GTR-GAMMA bootstrapping utilizing 1,000 replicates. The phylogenetic tree was subsequently edited and visualized with iTOL v.6.5.7 (29). Pairwise distances between isolates were calculated using Snp-Dists (30) following Gubbins correction. The resulting data were used to generate a heatmap depicting SNP distances across genomes.

### Statistical analysis

XLSTAT Version 2014.5.03 was used for statistical analysis. To determine non-random associations between two categorical variables, Fisher’s exact test was applied with significance set at *P* < 0.05.

## RESULTS AND DISCUSSION

### *C. neonatale* population characteristics

The features of the 48 newly sequenced and 12 publicly accessible genomes used in this study are listed in Table 1. The newly sequenced genomes were from strains isolated from independent neonates included in three distinct cohorts over three time periods (2008, 2011, and 2015–2016) (Fig. 1). A total of 13 strains were isolated from neonates with NEC and 38 from neonates without NEC. The *C. neonatale* strains were obtained from neonates as follows: 17 strains were obtained from the monocentric PREMAFLORA study (2008–2009); 17 strains (7 NEC cases and 10 controls) were obtained from the nationwide multicenter EPIFLORE study (2011); and 17 strains (6 NEC cases and 11 controls) were obtained from the nationwide multicenter ClosNEC (2015–2016). In addition, we included 12 publicly available genomes from databases (4, 5, 11, 12). Chronologically, 3 strains were recovered in 2002, 17 strains in 2008, 1 in 2010, 17 in 2011, 3 in 2012, 18 in 2015–2016, and 1 in 2021. The strains originated from 12 different NICUs across five French regions (Fig. S2).

**TABLE 1:** *Clostridium neonatale* genome data^*c*^

Strain ID	Year of isolation (reference)	Sample origin/NEC	Sequencing technology	Contigs/ genome close level	Contigs N50 (bp)	Contigs L50	Size genome (Kb)	Completeness/ contamination (%)	G+C (%)	CDS	tRNA	rRNA	Repeated regions (%)	Accession no.
250.09^*a*^	2002 (7, 11)	NEC	Pacbio/ HiSeq	1 Complete	4753394	1	4752	98.92/0.80	28.61	4356	85	33	7.79	ERS7257048
CC3_PB	2015 (3)	NEC	Pacbio/ HiSeq	6	5137519	1	5675	97.62/0.96	28.60	5505	91	32	15.33	ERS7257050
LF22	2011 (3)	Control	HiSeq	288	55509	32	5598	99.19/0.96	28.67	5602	78	11	10.55	ERS7257051
CB12	2011 (3)	NEC	HiSeq	83	119741	14	4612	99.19/0.80	28.38	4259	74	11	5.09	ERS7257049
C16	2015	Control	NextSeq	218	49190	33	5227	99.19/0.96	28.48	5134	67	6	6.78	ERS13471573
C191	2015	Control	NextSeq	251	63732	25	4973	99.38/0.96	28.74	4915	71	3	7.75	ERS13471572
C199	2015	NEC	NextSeq	216	59201	27	5281	99.19/0.96	28.48	5372	75	6	7.11	ERS13471571
C20	2015	NEC	NextSeq	223	43136	33	4770	93.54/0.96	28.68	4743	62	4	7.08	ERS13471570
C202	2015	Control	NextSeq	285	49224	34	5500	99.19/0.96	28.58	5556	71	6	7.94	ERS13471547
C25	2015	Control	NextSeq	245	53383	28	5180	99.19/0.96	28.65	5083	74	6	7.63	ERS13471548
C34	2016	Control	NextSeq	256	45820	32	5257	99.19/0.96	28.49	5172	69	6	7.12	ERS13471574
C38	2016	NEC	NextSeq	230	44913	25	3991	75.80/0.16	29.02	3941	53	5	7.28	ERS13471564
C40	2016	Control	NextSeq	305	36993	40	5321	99.19/0.96	28.66	5370	57	6	7.84	ERS13471565
C47	2016	NEC	NextSeq	266	46452	36	5408	99.19/0.96	28.58	5407	66	6	7.50	ERS13471567
C50	2016	NEC	NextSeq	244	53419	28	5331	99.19/0.96	28.59	5229	67	4	7.77	ERS13471569
C51	2016	NEC	NextSeq	307	37893	38	5304	99.19/0.96	28.66	5355	71	6	7.63	ERS13471568
C55	2016	Control	NextSeq	179	60825	25	4797	99.19/0.80	28.49	4614	70	4	6.17	ERS13471549
C58	2016	Control	NextSeq	273	47024	34	5428	99.19/0.80	28.60	5467	72	6	7.83	ERS13471566
C6	2015	Control	NextSeq	209	84139	20	4928	99.19/1.00	28.43	4807	78	6	6.47	ERS13471561
C7	2015	Control	NextSeq	267	56826	29	5334	99.19/0.96	28.60	5262	67	4	8.02	ERS13471562
E1	2011	NEC	HiSeq	408	38330	40	5349	99.19/1.21	28.52	5487	68	6	8.42	ERS13471587
C8.2	2015	Control	NextSeq	240	39749	37	4976	91.12/0.96	28.77	5029	63	5	7.84	ERS13471563
E18	2011	Control	HiSeq	129	97882	17	4827	99.19/1.70	28.37	4691	68	5	5.59	ERS13471576
E20	2011	Control	HiSeq	217	40923	34	4420	82.25/0.96	28.76	4362	58	5	6.90	ERS13471580
E24	2011	Control	HiSeq	113	87280	15	4738	99.19/0.80	28.40	4512	69	6	5.29	ERS13471578
E8	2011	NEC	HiSeq	216	57708	29	5334	99.19/0.96	28.51	5433	71	11	7.77	ERS13471575
E15	2011	Control	HiSeq	257	55166	30	5568	99.19/0.96	28.62	5692	67	11	9.41	ERS13471582
E11	2011	NEC	HiSeq	266	63678	26	5408	99.19/1.16	28.49	5514	74	10	9.06	ERS13471585
E10	2011	NEC	HiSeq	216	53364	30	5320	99.19/0.96	28.51	5424	73	11	7.50	ERS13471579
E7	2011	NEC	HiSeq	82	147824	10	4650	99.19/0.80	28.38	4444	77	11	4.86	ERS13471586
E16	2011	Control	HiSeq	112	96845	17	4940	99.19/0.80	28.58	4906	72	11	6.01	ERS13471583
E13	2011	Control	HiSeq	279	49779	34	5280	99.19/0.96	28.56	5403	72	11	8.11	ERS13471584
E31	2011	Control	HiSeq	217	55929	29	5328	99.19/0.96	28.51	5445	73	11	7.70	ERS13471588
E34	2011	Control	HiSeq	289	38727	41	5212	99.19/0.96	28.52	5198	58	10	6.90	ERS13471581
E32	2011	Control	HiSeq	182	69479	27	5271	99.19/0.96	28.47	5350	72	11	7.64	ERS13471577
PM104	2008	Control	NextSeq	313	67470	24	5349	99.19/1.16	28.48	5411	74	5	8.00	ERS13471589
PM106	2008	Control	NextSeq	314	66322	26	5341	99.19/1.16	28.49	5388	67	6	7.89	ERS13471590
PM111	2008	Control	NextSeq	318	56073	32	5213	99.19/1.16	28.56	5275	68	6	7.49	ERS13471591
PM116	2008	Control	NextSeq	303	62783	27	5338	99.19/1.16	28.50	5418	73	4	7.94	ERS13471592
PM120	2008	Control	NextSeq	363	68885	26	5370	99.19/1.16	28.49	5476	72	6	8.47	ERS13471593
PM124	2008	Control	NextSeq	295	60525	25	4811	95.69/1.16	28.84	4889	56	5	7.60	ERS13471594
PM68	2008	Control	NextSeq	341	60302	27	5335	99.19/1.16	28.50	5405	72	4	8	ERS13471595
PM77	2008	Control	NextSeq	104	124609	14	4716	99.19/0.80	28.34	4467	71	5	5.05	ERS13471596
PM81	2008	Control	NextSeq	118	87192	18	4663	99.19/0.84	28.35	4401	61	5	4.88	ERS13471597
PM101	2008	Control	NextSeq	240	66324	26	5359	99.19/1.16	28.62	5411	69	6	7.92	ERS13471598
PM102	2008	Control	NextSeq	295	66384	29	5317	99.19/1.16	28.50	5373	72	4	7.88	ERS13471599
PM103	2008	Control	NextSeq	300	33345	46	5421	99.19/1.16	28.65	5543	64	7	8.90	ERS13471600
PM74	2008	Control	NextSeq	550	55675	29	4744	91.93/1.16	28.67	4832	60	5	8.18	ERS13471601
PM42	2008	Control	NextSeq	291	90364	16	5744	98.92/1.00	28.42	4524	73	6	6.17	ERS13471602
PM84	2008	Control	NextSeq	135	81800	16	4377	95.96/0.80	28.56	4217	62	11	5.55	ERS13471603
PM54	2008	Control	NextSeq	309	52959	30	4936	95.96/1.16	28.78	5031	63	5	7.73	ERS13471604
PM53	2008	Control	NextSeq	240	63755	28	5408	99.19/1.16	28.61	5599	71	10	8.57	ERS13471605
NEC25	2011 (4)	NEC	MiSeq	3^*b*^	2546558	1	4739	97.58/0.80	28.67	3932	77	28	7.10	PRJEB26968
NEC26	2012 (4)	NEC	MiSeq	3^*b*^	2546622	1	4738	96.34/0.80	28.86	3808	77	28	7.08	PRJEB26973
NEC32	2012 (4)	NEC	MiSeq	3^*b*^	2546635	1	4738	97.58/0.80	28.75	3891	77	28	7.10	PRJEB27003
NEC86	2010 (4)	NEC	MiSeq	3^*b*^	2546783	1	4739	99.19/1.61	28.58	4304	77	28	7.84	PRJEB26949
C25-UICQ01	2012 (4)	Control	MiSeq	3^*b*^	2546563	1	4739	98.79/0.80	28.64	3937	77	28	7.09	PRJEB26947
LCDC99A005	2002 (2, 11)	NEC	MiSeq	7	3168417	1	4712	99.19/0.80	28.42	4454	69	11	6.85	AF275949
LCDC99A006	2002 (2, 11)	NEC	MiSeq	58	156000	10	4664	99.19/0.80	28.38	4405	81	10	5.45	PRJNA224116
Q4564	2021 (5)	NEC	MiSeq	844	96910	152	5263	97.81/1.77	28.64	5393	67	6	6.69	PRJNA714596

^*a*^ Corresponds to the reference strain LCDC99A005.

^*b*^ Contigs containing “N” stretches.

^*c*^ NEC, preterm infants with diagnosis of necrotizing enterocolitis; CDS, coding DNA sequences.

### Whole genome data

Regarding the sequencing data of the 60 *C. neonatale* genomes included in this study (Table 1), the genomes’ estimated size ranged from 3.9 Mb to 5.6 Mb, with an average length of 5 ± 0.36 Mb. The average genome G + C content was 28.57 ± 0.13%. The number of predicted protein-coding genes ranged from 3,808 to 5,692, with an average of 4,980 ± 533. Whole genome pairwise sequence comparisons revealed an ANI of 98.20% and 99.98% for the most distant and closest strains, respectively (Fig. S3). These data complement the previous findings of a comparative genomics study using nine *C. neonatale* genomes (12). The study reported a genome length ranging from 4.6 to 5.6 Mb (an average of 4.9 ± 0.40), a predicted number of protein-coding genes ranging from 4,259 to 5,505 (an average of 4,399 ± 684), an average G + C content of 28.64 ± 0.13%, and an ANI ranging from 98.41% to 99.83% for the most distant and the closest strains, respectively (12). In this study, the G + C content of *C. neonatale* genomes is comparable to that of other *Clostridium sensu stricto* members. Nevertheless, some variations exist in genome sizes, which range from 2.55 Mb for *C. novyi* to 6.53 Mb for *C. saccharoperbutylacetonicum*, and CDS numbers range from 2,601 for *C. tetani* to 5,533 for *C. saccharoperbutylacetonicum* (31).

In the current study, 58 out of the 60 analyzed genomes were classified as "high-quality" draft genomes. This definition is based on the minimum reporting standards for genomes, which require at least 90% completeness, a maximum of 5% contamination, the presence of 23S, 16S, and 5S rRNA genes, and a minimum of 18 tRNAs (32).

### *C. neonatale* cgMLST scheme and analysis

One of the most commonly used methods for bacterial typing in whole genome sequencing is the gene-by-gene cgMLST approach. The cgMLST method focuses on a wide range of CDSs present in most strains, which permits high discrimination and renders the system less prone to mutations such as insertions and deletions (33). Additionally, cgMLST does not require a specific reference genome, making it a suitable method for identifying potential clusters from samples of an entire species (34). In the current study, the wgMLST scheme was initially represented by a data set of 7,127 possible target loci identified from the 60 genomes that were analyzed (Fig. S4). After the filtering steps, 56 loci were found to be paralogous and were excluded. A genome quality test eliminated additional 4,721 loci. Ultimately, the cgMLST scheme included a total of 2,350 gene targets that were present in at least 95% of the genomes, meeting a well-defined cutoff (35 – 37). It was assumed that up to 5% of the loci in each strain may not be identified due to issues such as sequencing coverage or assembly or other factors associated with using draft genome (14) (Table S1).

The relatedness of the *C. neonatale* strains was investigated using the cgMLST scheme. Out of the 60 strains, five clades were identified as follows: clade I (*n* = 1, 2%), II (*n* = 9, 15%), III (*n* = 20, 33%), IV (*n* = 8, 13%), and V (*n* = 22, 37%) (Fig. 1). Clades III and V had the highest strain diversity when considering the period of isolation. The strain LF22 was the only representative of clade I, suggesting higher genetic diversity in this strain compared to that in others. The reference strain 250.09 as well as two strains isolated during the same outbreak (LCDC99A005 and LCDC99A006) (2 – 5, 11) belonged to clade II. Additionally, six strains isolated from different regions in 2008 or 2011 also belonged to clade II (Fig. 1). Some clades included mostly strains isolated from the same period (or cohort), particularly clade III in 2008, corresponding to the monocentric cohort PREMAFLORA. This was also observed for other clades and time periods, but to a lesser extent. Within each clade, certain genomes are organized in tight (near-clonal) groups, suggesting the same clone spread in the same NICU. This is in agreement with the draft genome-based phylogeny and core-genome analysis of a small number of *C. neonatale* isolates (*n* = 5), which report clonality among strains within the same NICU (4). However, some strains were also distributed independently of the isolation period (Fig. 1). Geographically, similar findings were obtained. Taken together, our results support the distribution of the *C. neonatale* strain independent of spatial and temporal clustering.

Compared to the multilocus sequencing analysis scheme previously used to study the phylogenetic relationship of four *C. neonatale* strains (7), our results placed three of these strains (LF22, PM53), and 250.09) into distinct clusters, providing a better resolution for distinguishing between the genetic relatedness of the strains.

### cgSNP analysis and comparison with cgMLST

Alternatively, the genetic relatedness of strains can be determined using the SNP typing approach, which identifies single nucleotide polymorphisms (SNPs) that differ between strains. SNPs are detected by mapping sequence reads against a closely related reference genome and recording nucleotide differences (13). In particular, the handling of recombination events differs between cgMLST and SNP alignments. The cgMLST method collapses recombination regions containing a high density of SNPs into fewer allelic changes (17), while they can be filtered in the SNP alignment.

**Fig 2:**
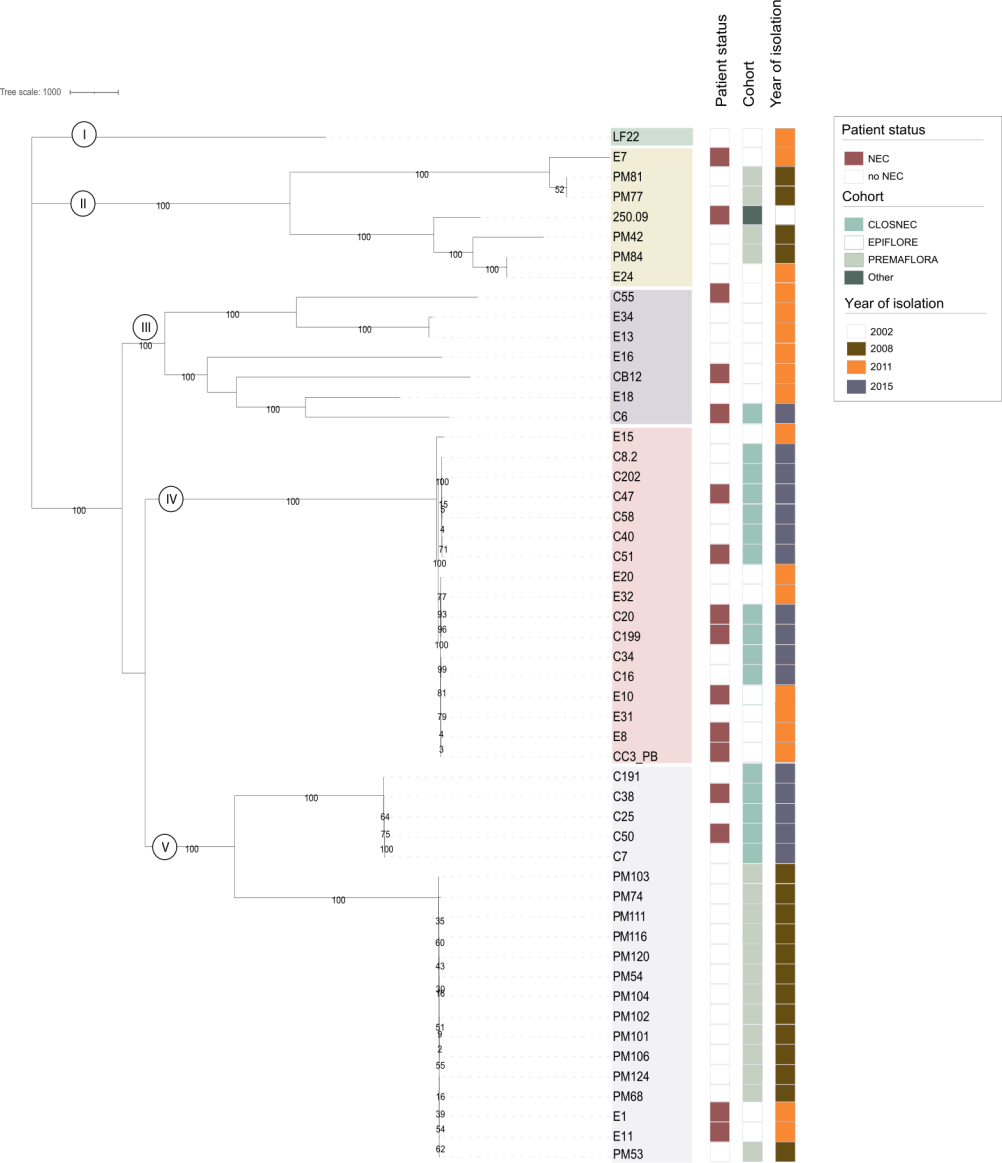
Maximum likelihood cgSNP phylogenetic tree (bootstrapping: 1,000 replicates) based on the 31,248 cgSNPs after removal of the predicted recombinant regions. Strain ID, patient status (NEC cases or controls), cohort, and year of isolation are given for each strain. The *C. neonatale* type strain 250.09 is the reference strain (4, 5).

Using *C. neonatale* 250.09 as the reference genome, the cgSNP calling step resulted in an alignment of 31,248 SNPs subsequent to the removal of the predicted recombinant regions, based on the raw data availability of 52 genomes (Table S2). The maximum likelihood phylogenetic tree of cgSNP enabled the clustering of strains within the five identical clades as cgMLST (Fig. 2). This was confirmed by the heatmap showing pairwise SNP distances across the strain genomes differing by at least one and at most 12,826 SNPs and distributed into the five clades (Fig. S5). The cgMLST results indicated that some strains were clonal and associated with isolation from the same period or cohort. However, clonal strains were also observed independently of clustering and periods or cohorts, suggesting possible clone dissemination.

**Fig 3:**
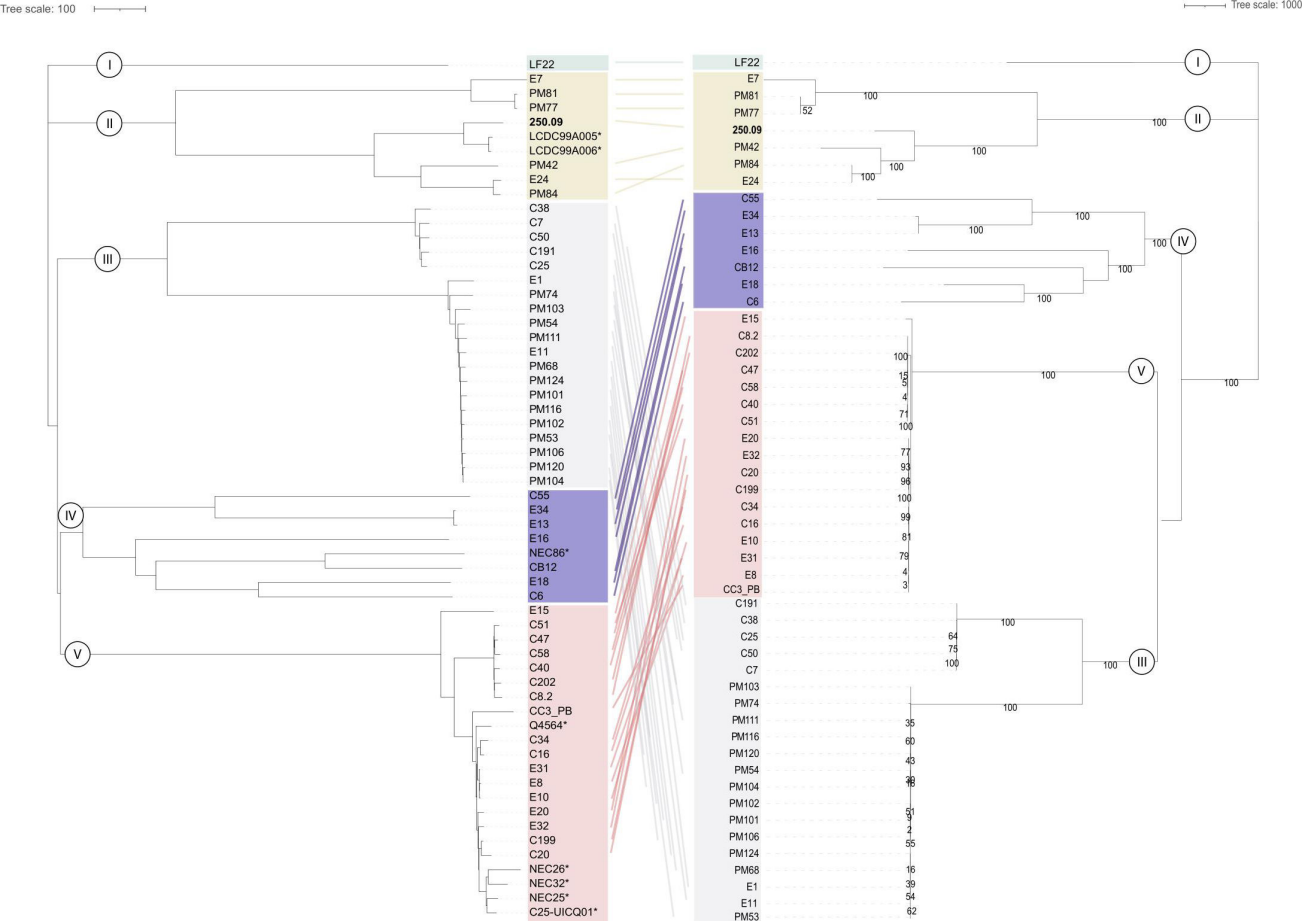
Comparison of the cgMLST neighbor-joining tree (left) and the cgSNP maximum likelihood tree (right). The *C. neonatale* type strain 250.09 is the reference strain (bold). Connecting lines indicate changed positions between the two trees. (*) Strains from other studies.

The comparison of the cgMLST and cgSNP-based phylogenetic analyses of the 60 genomes is presented in Fig. 3. The phylogenetic tree for cgSNPs confirmed our cgMLST results. Although differences in topology and strain distance were noticed in clades III, IV, and V, the two methods produced similar results indicating equal discriminatory power. This is consistent with other studies that showed a high level of agreement between the cgMLST and cgSNP approaches, resulting in comparable levels of differentiation and relatedness (13, 33, 35).

### cgMLST- and cgSNP-based analyses of *C. neonatale* strains isolated from patients with or without NEC

**Fig 4:**
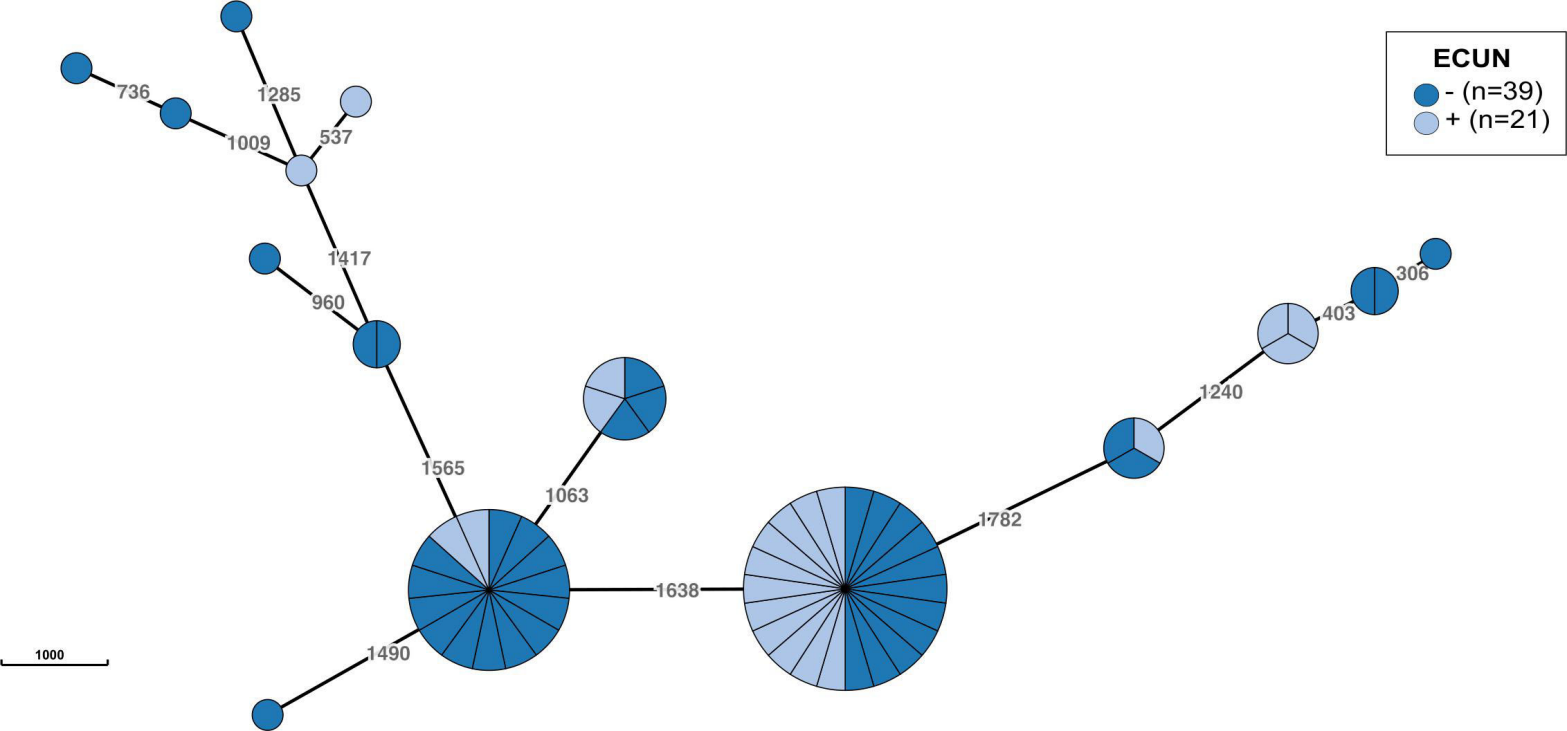
Minimum-spanning tree based on the cgMLST allelic profiles of the 60 *C. neonatale* strains. Each circle represents an allelic profile based on the sequence analysis of the 2,350 target genes of the cgMLST scheme. The numbers on the connecting lines indicate the number of target genes with different alleles. The colors represent the strains according to the NEC status of the patient.

*C. neonatale* is one of the species found in the stool samples of preterm neonates with NEC (3 – 5, 8). However, there are limited data available on the epidemiological surveillance and clinical monitoring of this potential opportunistic pathogen. As we have demonstrated that both cgMLST- and SNP-based methods yield similar results, a minimum-spanning phylogenetic tree based on cgMLST was generated to compare the distribution of the strains isolated from NEC cases and control patients (Fig. 4). Our results showed a distribution of strains among 15 cgMLST types. We observed a dominance of two cgMLST types that differed by 1,638 alleles and encompassed 62% (*n* = 37) of the strains (Fig. 4). The distribution of the strains was independent of their isolating group (i.e., NEC cases vs controls). Regarding the period of isolation, there were no significant differences observed between *C. neonatale* strains and NEC (*P* = 0.74). Previously, a possible existence of NICU clones has been proposed without a relationship to the occurrence of NEC in preterm neonates (4). In this study, no significant differences were observed based on the geographical location (*P* = 0.29). With regard to NEC, the clade distribution of *C. neonatale* strains did not correlate with the occurrence of NEC (*P* = 1). Altogether, our findings did not establish a connection between a particular genetic type of *C. neonatale* and the patient’s NEC status. This suggests that additional factors associated with host receptivity or gut microbiota could be implicated. Besides, the similarity between control and NEC-associated *C. neonatale* in clusters presupposes the existence of asymptomatic carriage.

### Strengths and limitations

One limitation of this study is the lack of strain diversity as the analysis only included strains from neonates. However, it is notable that the number of *C. neonatale* strains and available genomes were limited. In addition, to our knowledge, the natural reservoir for *C. neonatale* remains unknown as strains from adult humans, animals, and the environment are not currently available. Although a total of 12 NICUs participated in the study, the low number of patients included in some NICUs hindered our regional analysis due to low statistical power. The strength of this study is that it utilizes the well-characterized 250.09 reference strain genome as a seed genome as well as the largest, well-characterized, and diverse collection of *C. neonatale* strains obtained from different spatio-temporal settings with ≥95% cgMLST targets.

### Conclusions

Little information is available about *C. neonatale,* one of the potential pathogens associated with NEC. Whole genome sequencing typing methods were applied to evaluate the phylogenetic and epidemiological links among a unique collection of clinical *C. neonatale* strains isolated in different spatio-temporal settings. We developed and established a potentially stable cgMLST scheme for *ad hoc* usage, providing an applicable and discriminatory typing method at the strain level. We have demonstrated that both cgMLST and coreSNP exhibit similar discriminatory abilities. This study sheds light on the epidemiology and population dynamics of *C. neonatale*. It offers new insights into the distribution of *C. neonatale* strain clonal complexes among NEC patients. The proposed cgMLST scheme will contribute to the study of the molecular dynamics of *C. neonatale* strains, providing information for local and global clinical surveillance of this opportunistic pathogen.

## Data Availability

Sequence read files for the 48 isolates included in the present study have been deposited in SRA and are accessible in Genbank through NCBI BioSample accession numbers and whole genome shotgun projects as follows: PRJEB56034 (PREMAFLORA study), PRJEB56033 (ClosNEC study), and PRJEB53861 (EPIFLORE study). Genome data are presented in Table 1. The proposed wgMLST schema is available at https://chewbbaca.online/.

## SUPPLEMENTAL MATERIAL

Supplemental file 1 spectrum.02766-23-s0001.pdf Fig. S1 to S5.

Supplemental file 2 spectrum.02766-23-s0002.xlsx Table S1 (cgMLST profiles).

ASM does not own the copyrights to Supplemental Material that may be linked to, or accessed through, an article. The authors have granted ASM a non-exclusive, world-wide license to publish the Supplemental Material files. Please contact the corresponding author directly for reuse.
