# Supplementary material for: Core-genome multilocus sequence typing and core-SNP analysis of Clostridium neonatale strains isolated in different spatio-temporal settings
Source: Microbiol Spectr. 2023 Nov 1;11(6):e02766-23. doi: 10.1128/spectrum.02766-23 (PMC10714970; doi:10.1128/spectrum.02766-23)
Supplement: Supplemental file 1 — Fig. S1 to S5. [file spectrum.02766-23-s0001.pdf]

Supplemental Material

A

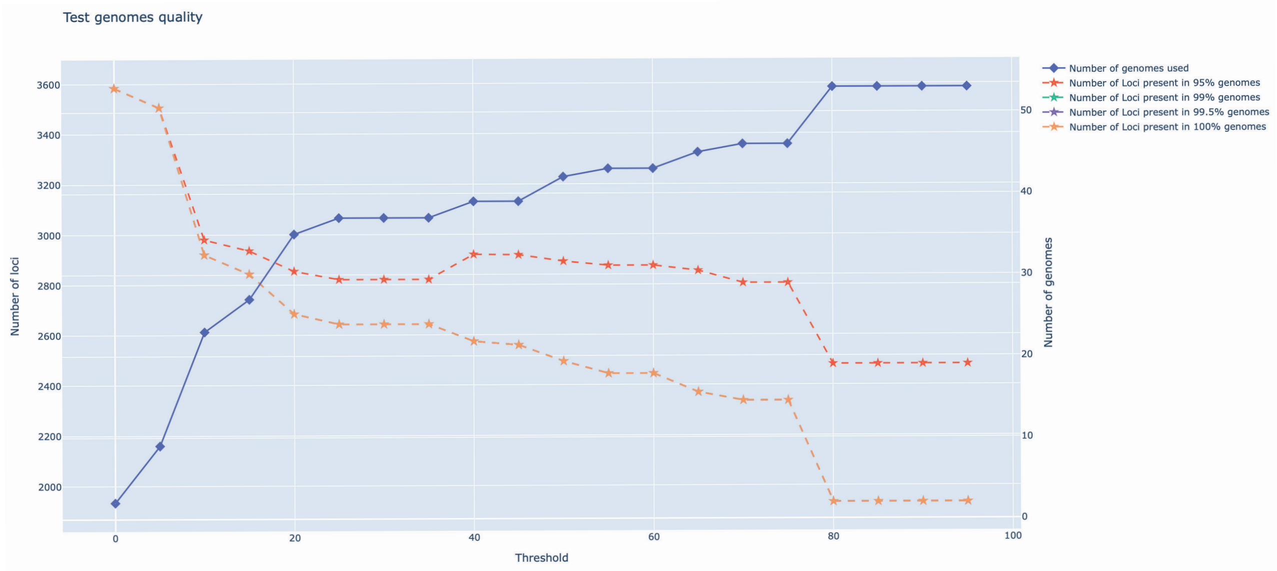

B

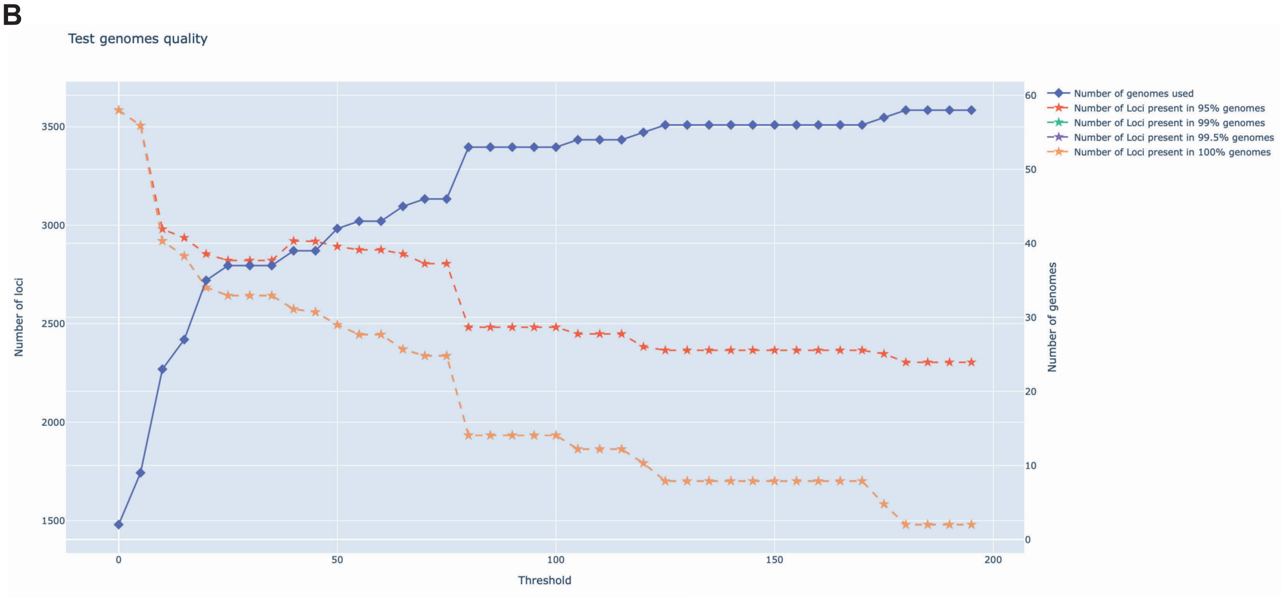

Supplementary Figure S1. Test quality of the wgMLST scheme. Number of loci and number of genomes in each exclusion threshold level of 100 **(A)** and 200 **(B)** based on the sixty strain genomes. Results are as follows: 2,482 loci and 53 retained genomes for the 100 threshold; 2,304 loci and 58 retained genomes for the 200 threshold. The number of loci found in 95%, 99%, 99.5%, and 100% of the genomes is shown.

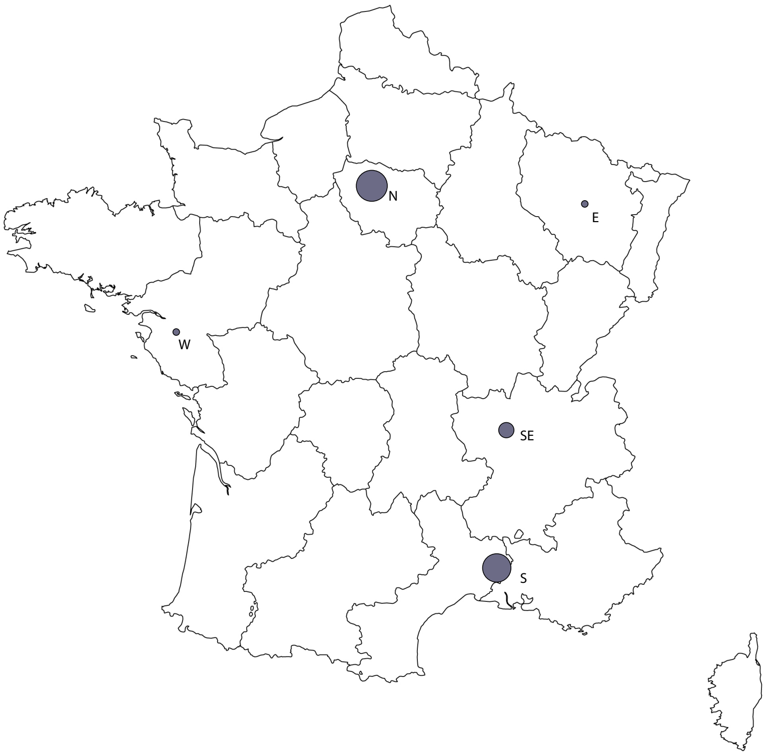

Strain ID	Regions
CC3_PB	S
LF22	W
CB12	W
C16	SE
C191	S
C199	S
C20	S
C202	N
C25	N
C34	SE
C38	N
C40	N
C47	N
C50	N
C51	N
C55	E
C58	N
C6	N
C7	N
E1	N
C8.2	N
E18	ND
E20	S
E24	SE
E8	S
E15	ND
E11	N
E10	S
E7	N
E16	ND
E13	ND
E31	ND
E34	ND
E32	S
PM104	N
PM106	N
PM111	N
PM116	N
PM120	N
PM124	N
PM68	N
PM77	N
PM81	N
PM101	N
PM102	N
PM103	N
PM74	N
PM42	N
PM84	N
PM54	N
PM53	N
NEC25	S
NEC26	S
NEC32	S
NEC86	S
C25-UICQ01	S
Q4564	S

Supplementary Figure S2. Schematic representation of the 5 different regions of France indicating the localization of NICUs associated with *C. neonatale* strains ID. ND: not determined.

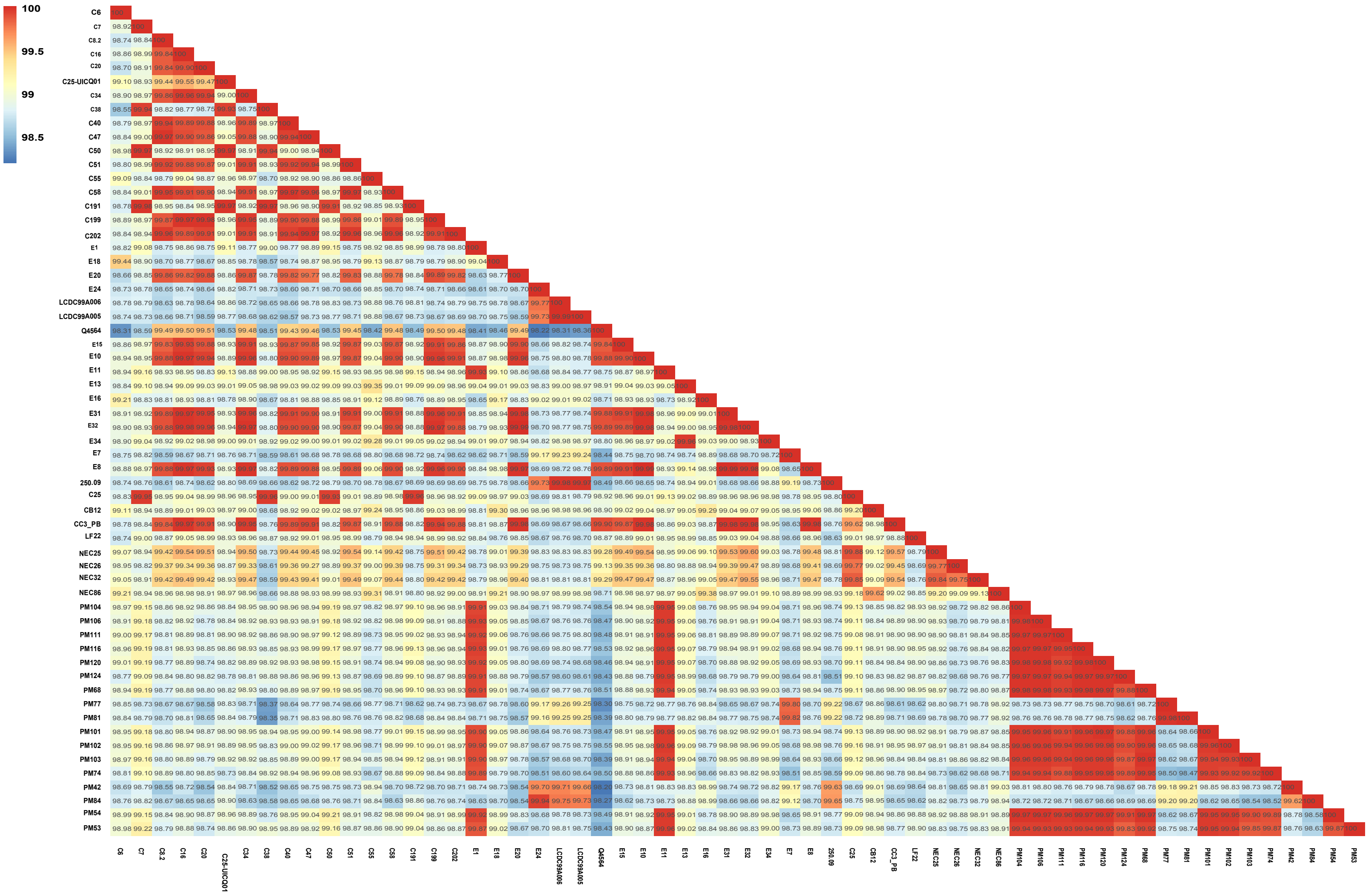

Supplementary Figure S3. Heatmap showing the level of genome similarity based on the average nucleotide identity (ANI). The heatmap was created using the high similarity (dark red) and low similarity (blue) of coding DNA sequences (CDS) derived from the sixty C. neonatale genomes.

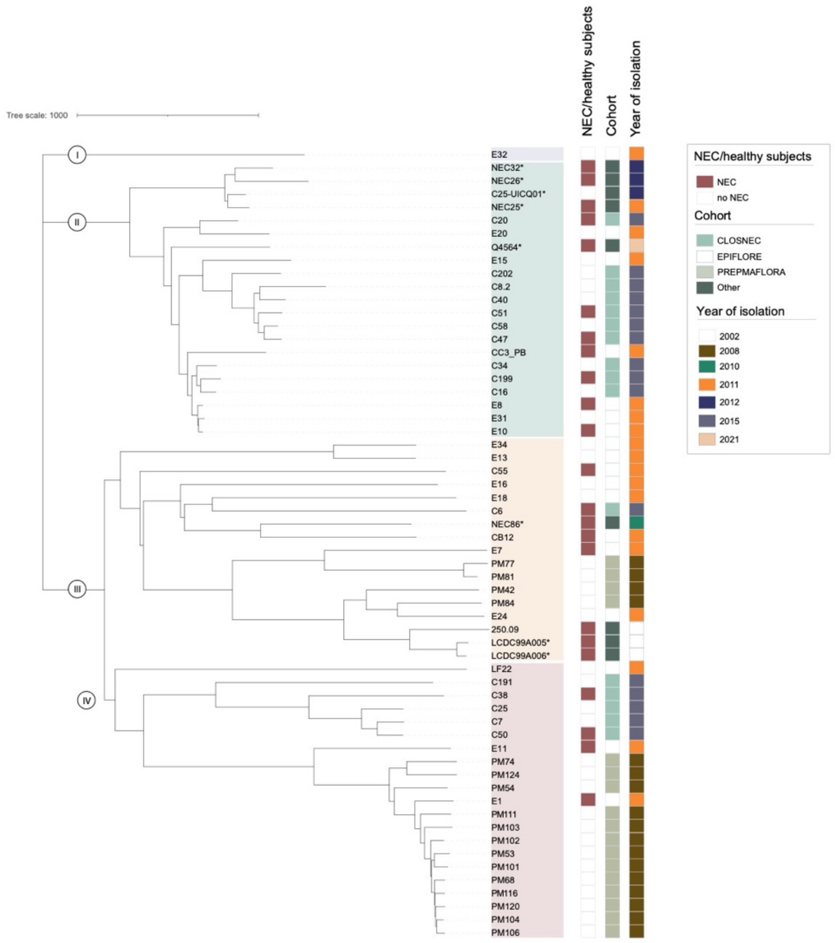

Supplementary Figure S4. Neighbor-joining wgMLST phylogenetic tree of sixty *C. neonatale* strains. For each strain, the strain ID, patient status (NEC or controls), cohort, and year of isolation are provided. The reference strain is 250.09, the *C. neonatale* type strain. Tree was determined using the chewBACCA software and was created with GrapeTree software version 1.5. (*) Strains from other studies (5, 29, 30).

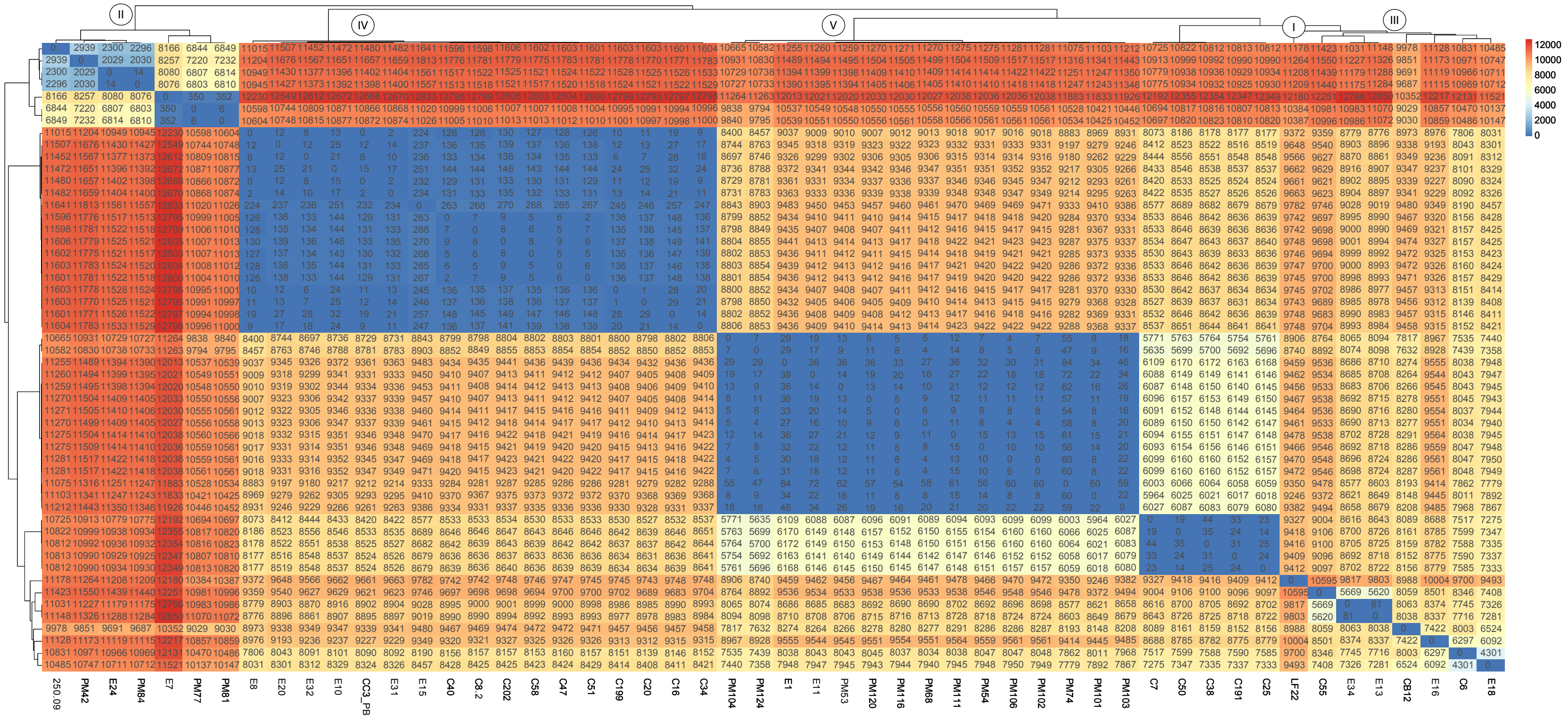

Supplementary Figure S5. Pairwise distances between non-recombinant SNPs of the sixty C. neonatale strains, based on the cgSNP. Each cell of the heatmap shows the number of different SNPs used for measuring genetic distances across the strains. Strain are clustered according to the pairwise differences distance matrix.
